# Two Kinds of “Woke”? Psychometric Validation of the Critical Right Scale and Revised Critical Social Justice Attitudes Scale

**DOI:** 10.1111/sjop.70070

**Published:** 2026-01-10

**Authors:** Oskari Lahtinen

**Affiliations:** ^1^ INVEST Flagship/Department of Psychology University of Turku Turku Finland

**Keywords:** critical right, critical right scale, critical social justice, critical social justice attitudes, woke right

## Abstract

This study developed and validated the Critical Right Scale (CRS) to measure emerging critical right attitudes and revised the Critical Social Justice Attitudes Scale (CSJAS‐R), replicating its psychometric evaluation. A nationwide convenience sample of Finnish adults (*n* = 626) completed an online survey. Item screening used exploratory factor analysis with oblique rotation and loading and residual correlation criteria. Confirmatory factor analysis (CFA) and measurement invariance testing were conducted in *lavaan* using full information maximum likelihood. The final CRS consisted of five items with high reliability (*α* = 0.89, *ω* = 0.90) and good model fit in both male and female subsamples, with pooled‐sample residual misfit judged minor given subgroup results. The CSJAS‐R comprised six items with strong reliability (*α* = 0.88, *ω* = 0.89) and excellent fit. Both scales met configural and metric invariance; partial scalar invariance was achieved after freeing a small number of item intercepts. CRS scores were strongly associated with right‐wing and conservative self‐placement with higher scores concentrated among Finns Party and Christian Democrat voters, and weakly linked to perceived oppression. CSJAS‐R scores were strongly associated with left‐wing and liberal self‐placement with higher scores concentrated among Left Alliance and Greens voters, and had a small‐to‐moderate association with justification of political violence. CRS and CSJAS‐R were strongly negatively correlated (*r* = −0.62), indicating divergent validity. Both CRS and CSJAS‐R demonstrated strong psychometric properties and distinct ideological profiles, providing validated tools for studying political attitude structures at opposing ends of the ideological spectrum.

## Key Points

- The Critical Right Scale (CRS; 5 items) and the revised Critical Social Justice Attitudes Scale (CSJAS‐R; 6 items) both showed strong reliability and good‐to‐excellent CFA fit in a Finnish adult sample (*n* = 626).
- Both scales demonstrated measurement invariance across gender at the configural and metric levels, with partial scalar invariance after freeing a small number of intercepts, supporting meaningful group comparisons.
- CRS and CSJAS‐R mapped onto opposing ideological poles: CRS aligned strongly with right‐wing and conservative self‐placement and clustered among Finns Party and Christian Democrat voters; CSJAS‐R aligned strongly with left‐wing and liberal self‐placement and clustered among Left Alliance and Greens voters.
- The two constructs showed strong divergent validity: CRS and CSJAS‐R were strongly negatively correlated (*r* = −0.62), indicating two distinct “critical” ideological orientations rather than one general factor.
- CSJAS‐R (but not CRS) was moderately associated with justification of political violence, whereas CRS was only weakly linked to perceived oppression and showed no meaningful association with happiness.

During the late 2010s, many Western societies saw the rise of an ideology sometimes referred to as “critical social justice”, which more people refer to, sometimes pejoratively, as “wokeness”. This worldview emphasizes the primacy of identity groups (e.g., black/white, gay/straight, trans/cis) and interprets social dynamics in terms of systemic oppression and privilege (e.g., policing as structurally racist; exclusion of trans women from female spaces as unjust) (Mounk 2023; Sensoy and DiAngelo 2017). It has also promoted regulation of expression to avoid “problematic” speech or action, including “calling out” individuals for perceived transgressions. In moral foundation theory terms, its central tenets could be argued to be care and fairness with a hint of authority (Graham et al. 2013).

Although its cultural peak may have occurred around the George Floyd protests in 2020, “wokeness” remains influential, particularly on the political left. In Lahtinen (2024), I developed the Critical Social Justice Attitudes Scale (CSJAS) to measure these attitudes. Across two Finnish samples, support for CSJ attitudes was strongly gendered—nearly 60% of women endorsed them, compared to 15% of men—and negatively correlated with well‐being, though no more strongly than general left‐wing affiliation. As left‐wing political affiliation is itself more common in women, the direction of the gender divide is understandable, even if the numbers are stark. The present study examines the CSJAS to replicate and update its psychometric evaluation.

Recent discourse suggests that a parallel phenomenon may be emerging on the political right. In 2022, *The Gospel Coalition* described Stephen Wolfe's *The Case for Christian Nationalism* as part of “The Rise of Right‐Wing Wokeism” (DeYoung 2022). By 2025, the term “woke right” appeared in mainstream outlets including *The New York Times*, *The Atlantic*, and *Financial Times* (Chatterton‐Williams 2025; Kelly 2025; McWhorter 2025b). Like its left‐wing counterpart, this ideology centers identity‐based grievance, inverted oppressor–oppressed dynamics (e.g., DEI as discrimination; pro‐Palestinian protests as antisemitic), and speech regulation (e.g., language restrictions under the Trump administration).

As on the left, the term “woke right” is often used pejoratively in popular discourse. However commentators have noted an emergent ideology on the right that seems worthy of study. I adopt the term *critical right* to describe this cluster of attitudes. Though not a critical theory in the traditional sense, it is called critical because it centers identity‐based grievance and identifies a systemic oppressor. It is an inversion of a critical theory in its attempt to preserve and restore majoritarian hierarchies and the nation state.

Critical right is here operationalized via three proposed dimensions. *Identitarian grievance* captures group‐based victimhood claims involving identities such as white, Western, Christian, male, or heterosexual, based on perceived status threat (Mutz 2018). The perceived oppressors are institutions and liberal elites which use their power to devalue and dispossess the majority in‐group. *Postmodern conservatism* refers to a form of conservatism informed by postmodern critiques of modernity (Lawler 2002; McManus 2019), marked by cultural resentment, affect‐driven politics, skepticism toward objectivity, and performative discourse.

Critical right is postmodern in the sense that it is skeptical toward opposing narratives (liberalism, globalism), specifically targets (“woke” or politically correct) language, and places power above objectivity. It should however be noted that it is not postmodern in the sense that it ends up creating a new metanarrative of national unity. *Authoritarian nationalism* fuses strong national identity with centralized authority, low tolerance for pluralism, and skepticism toward international institutions, drawing on a longer intellectual lineage (e.g., Buchanan 2011; Schmitt 1932). This fusion is psychologically rooted in a desire for collective order and group dominance, which are the fundamental motivational goals driving ideological attitudes toward hierarchy and exclusion (Duckitt and Sibley 2010). Thus, this dimension captures the Critical Right's ultimate political aim of using the state to restore majoritarian hierarchy and secure national unity. Viewed through moral foundation theory, the critical right places foremost emphasis on authority and loyalty (Graham et al. 2013). The Critical Right Scale (CRS) introduced here is designed to measure these dimensions while mirroring the logic of the CSJAS in emphasizing identity group hierarchies and regulation of speech. In contrast to this point of convergence, CRS and CSJAS diverge sharply in which identities they center and where they see oppression as coming from.

This study's primary aim is to develop and validate the CRS; the secondary aim is to replicate the psychometric evaluation of the CSJAS, after including new items in the candidate item pool and refining language in some items. The study will test whether it can be improved with refined items. Divergent validity for both CSJAS‐R and CRS will be assessed primarily via their assumed negative intercorrelation, as the two scales are hypothesized to measure ideologically opposing constructs. Convergent validity for both scales will be tested through their predicted high correlations with the Left–Right and Liberal‐Conservative political axes, and their concentration among specific party constituencies. The CSJAS‐R derives an additional, more direct measure of convergent validity from a self‐evaluated “wokeness” question, whereas an equivalent measure does not exist for the CRS. Partial convergent validity for CRS will rely on whether it aligns with right‐wing and conservative self‐placement and party affiliation. Previous research on CSJAS found a weak connection between CSJAS and (un)happiness, and associations with happiness will thus be examined for both CRS and CSJAS. In addition, a host of contextual variables were included in exploratory analyzes to assess correlations between CRS and CJSAS on the one hand and various attitudinal and value‐based propositions on the other.

## 1 Method

A data file and a codebook for the study have been posted online on Open Science Framework and are found here: https://osf.io/9ux6m/files/osfstorage

### 1.1 Participants and Procedure

The study was conducted as an online survey, which was administered via the Webropol online survey service. Data collection began on 10th of March, 2025 and ended on 9th of June 2025. The respondents were Finnish speakers who responded to an online ad that was distributed via several online channels and through social media of (predominantly right‐wing) Finnish politicians, as well as private sharing of the survey link.

The study had a sample of *n* = 626 participants. 43.3% of participants were female, 50.5% male, 2.6% classified themselves as “other”, 0.3% as transwomen, 0.2% as transmen, and 3.2% declined to answer. The modal age group was 40–49 years. Age categories were 18–24 (61 participants), 25–29 (60), 30–39 (101), 40–49 (149), 50–59 (121), 60–69 (74), 70–79 (42), 80+ (3). Mode for education was master's (29.4% of respondents), whereas 2.7% had lower secondary school as their highest education, 13.7% vocational degree, 11.3% student exam, 18.8% applied university degree, 12.3% bachelor's, 1.3% licentiate, 6.9% doctoral degree, 1.1% other, and 2.4% N/A. 18.5% of the sample were students, 6.9% taught or did research at a university, 4.3% had another job at a university, 68.5% were neither students nor staff at a university, and 1.8% N/A.

The study attempted a moderate oversampling of right‐wing participants by distributing the survey link via three politicians/party workers in the National Coalition and Finns parties. 26.5% (vs. 20.2% in an April–May poll published by Finnish Broadcasting Company) of the participants reported they vote for the National Coalition party, 15.8% (vs. 11.4%) for the Finns Party, 13.1% (vs. 9.4%) for the Left Alliance, 10.9% (vs. 7.9%) for the Greens, 8.8% (vs. 3.5%) for the Christian Democrats, 5.4% (vs. 25.3%) for the Social Democratic Party, 1.9% (vs. 15.6%) for the Center Party, 1.4% (vs. 3.9%) for the Swedish People's Party, 1.9% (vs. 1.9%) for another party, and 2.1% said they don't vote (Pelkonen 2025).

For additional context, a high‐profile Finnish demographic study, which collected data during the same time as the present study, was released by EVA (2025) in the summer of 2025. Their representative sample of Finnish voters had 49% responding they were right wing, 19% responding they were in the political center and 31% responding they were left wing. In the present study corresponding numbers were 51.5%, 16.2%, and 33.0%. The EVA sample was more liberal (53% of respondents; 29.8% in this sample) and slightly less conservative (24%; 27.5% in this sample). The present study had 42.7% indicating they were in between liberal and conservative.

### 1.2 Critical Right Scale Creation

Candidate items for the scale were formulated based on common statements found in political speeches, YouTube channels, television segments, social media, podcasts, and forums of individuals supportive of right‐wing identity politics, authoritarian nationalism, or postmodern conservatism, as defined in the study. An initial item pool of 18 was designed, with the goal of refining it to a unidimensional scale of 5–10 items. Two pilot testers with graduate degrees in social sciences and psychology evaluated the items and gave feedback on their development.

### 1.3 Measures

#### 1.3.1 Critical Right Attitudes

Critical right attitudes were measured with 18 candidate items for the Critical Right Scale. All items were devised by the author, using feedback from pilot testers to fine‐tune details in phrasing. Answer options for these items were 1 = “completely disagree”, 2 = “somewhat disagree”, 3 = “neither agree nor disagree”, 4 = “somewhat agree”, and 5 = “completely agree”. See Table 2 for a full list of items and Table 3 for a full list of the original Finnish items.

#### 1.3.2 Critical Social Justice Attitudes

Critical social justice attitudes were measured with the 7 items from the original Critical Social Justice Attitudes Scale (CSJAS) plus another 4 candidate items. Three candidate items were from a previous study and one about structural racism was devised for this study (*blinded*). Some items were slightly revised for clarity (see Tables 2 and 3 for complete wordings). Scale items have answer options ranging from 1 = “completely disagree” to 5 = “completely agree”. The original CSJAS was constructed and validated in an earlier study (Lahtinen 2024). The original scale had good reliability in the present study sample: *α* = 0.85, *ω* = 0.86.

#### 1.3.3 Happiness

Happiness was measured with a global happiness item from UN's World Happiness Report, where participants are asked to rate their quality of life on a scale from 0 to 10 (Helliwell et al. 2020).

#### 1.3.4 Additional Items

In addition to the 7 CSJAS items, several other context questions were asked from the participants. Participants rated 17 items assessing their views on institutions (e.g., European Union), ideologies (e.g., intersectional feminism), movements (e.g., Extinction Rebellion), world leaders (e.g., Donald Trump), and ongoing wars (e.g., Gaza war). Most questions were phrased “I appreciate [institution/ideology/movement/leader/event]” with answer options from 1 = “not at all”, 2 = “not much”, 3 = “neither little nor a lot”, 4 = “quite much”, and 5 = “a lot”. There were also items assessing whether participants had experienced oppression themselves, whether they had an inner or outer locus of control (“Other people or structures are more responsible for my well‐being than I myself am.”), whether they believed political violence was justified, and whether they thought violence against women could ever be justified. There was also a global critical social justice attitude item, which asked participants to self‐assess their critical social justice orientation (“If my friend called me “woke*” in good faith, I would agree with them, regardless of whether I approve of the term or not*.”). One item asked about how participants thought the war in Gaza should end (1 = “Two state solution”, 2 = “One state: Palestine”, 3 = “One state: Israel”, 4 = “Other solution”).

### 1.4 Compliance With Ethical Standards

All procedures performed involving human participants were in accordance with the ethical standards of the institutional and/or national research committee and with the 1964 Helsinki declaration and its later amendments or comparable ethical standards. All participants were adults and informed consent was obtained from all individual participants included in the study. Before answering the survey, participants were presented with the following consent message: “The answers to this survey will be used in a study on political attitudes. Your answers will be saved and analyzed anonymously in the study. By continuing to fill out the survey you indicate you have understood the above and give your consent to your answers being used as anonymous research data.” ChatGPT was used in editing the language in this manuscript and for advice in statistical analyzes.

### 1.5 Analysis Plan

Interitem correlations were to be calculated for all the 18 candidate items for the Critical Right Scale (CRS). Exploratory factor analyzes (EFA, in SPSS) would be used to evaluate the factor structure of the scale and how excluding some of the worst‐performing items would affect the factor structure and reliability of the scale. The factor solution would be obtained using principal axis factoring and corroborated using parallel analysis. Rotation method would be oblique (“direct oblimin” in SPSS) to allow correlations between factors. Loadings were expected to be above 0.50 and items below that threshold would be discarded from the model. To improve CFA fit, standardized residual correlations for an item would be used as exclusion criteria for items, with ideally no absolute values of *r* > 2.0. Item removal order would be determined by the number of violating residual correlations for that item, while considering the scale's conceptual integrity. If feasible, the scale was expected to have reliability over *ω* = 0.80 and explain at a minimum ≥ 50.0% of variance. Finally, confirmatory factor analyzes (CFA, in R) would be used to evaluate model fit for the scale, with missing values dealt by FIML. The scale was expected to have model fit of CFI = 0.95 or better, SRMR < 0.05, and RMSEA = 0.06 or lower, and to be of economical length, in the range of 5–10 items. Measurement invariance would then be tested for key grouping variables (e.g., sex, political orientation). This would involve sequentially fitting configural, metric, and scalar invariance models in lavaan, using ΔCFI ≤ 0.01 and ΔRMSEA ≤ 0.015 as thresholds for invariance (Chen 2007). If full scalar invariance was not met, partial scalar invariance would be tested by freeing the intercepts of noninvariant items based on modification indices. Study subpopulations (e.g., National Coalition Party voters, Finns Party voters, Christian Democrat voters, and nonvoters) would then be compared to each other in terms of critical right attitudes, opinions, and other variables. An identical procedure would then be run for the original CSJAS items and 4 additional items.

## 2 Results

### 2.1 Scale Reliabilities, EFAs, and CFAs

#### 2.1.1 CRS

All 18 candidate items (CRS1‐18) together had *α* = 0.92. The first EFA was run for 18 items. The items had KMO = 0.95 and Bartlett's sphericity Χ^2^ (153) = 4190.3, *p* < 0.001, indicating an excellent starting point for factor analyzes, and these indices remained good‐to‐excellent throughout analyzes. 18‐item EFA indicated three potential factors explaining 42.2%, 3.6%, and 2.6% of variance (eigenvalues: 8.05, 1.32, and 1.10). However, only two items had their strongest loading on the second factor and third factor (CRS16 on the second, CRS17 on the third). The weakest loading item (CRS18) was removed, which resulted in a three‐factor solution with 43.6%, 3.5%, and 2.8% of variance explained (eigenvalues 7.8, 1.3, and 1.1). Removing the next weakest item (CRS17) resulted in a two‐factor solution with 46.1% and 3.5% of variance explained by the factors (7.8 and 1.2). Removing the weakest loading item (CRS16) resulted in a unifactorial model with 49.0% of variance explained (eigenvalue 7.8). Possible problems were identified with some remaining scale items. CRS15 was deemed to be too context dependent to generalize across subpopulations. CRS14 seemed to measure fiscal conservatism more than authoritarian nationalism and was similarly excluded. After the exclusion of CRS14‐15, CRS13 loaded under 0.50 and was also removed. The remaining 12 items had loadings between 0.61–0.82 and *α* = 0.93 and *ω* = 0.93, were then transported over to CFA.

Confirmatory factor analysis for a 12‐item, single‐factor scale indicated mediocre fit: CFI = 0.94, TLI = 0.92, SRMR = 0.04, χ^2^(54, 623) = 323.1, *p* < 0.001, RMSEA = 0.09. Residual correlations were then inspected to improve model fit. Five items (CRS4, CRS9–12) were removed due to multiple standardized residual correlations exceeding |2.0|. Of the remaining seven items (CRS1–3, CRS5‐8), CRS8 was removed to improve conceptual fit, and CRS5 for an above‐threshold residual correlation. This yielded a well‐fitting model with one residual correlation > |2.0|. Removing CRS7 with the conceptually better‐fitting CRS4 improved the speech‐regulation facet, but subsequent testing showed the model still fit poorly for men. To address this, CRS6 was replaced with CRS5, producing good fit in both male and female subsamples: CFI = 0.996, TLI = 0.992, SRMR = 0.020, χ^2^(5, 316) = 7.33, *p* = 0.197, RMSEA = 0.038 (men); CFI = 0.997, TLI = 0.994, SRMR = 0.019, χ^2^(5, 271) = 6.28, *p* = 0.280, RMSEA = 0.031 (women). However, in the pooled sample this solution retained one standardized residual correlation close to 3.0 (CRS4–CRS5 = 2.94) and two slightly above |2.0| (CRS1–CRS2 = 2.17, CRS1–CRS4 = −2.26). Model fit was CFI = 0.992, TLI = 0.992, SRMR = 0.019, χ^2^(5, 623) = 13.28, *p* = 0.021, RMSEA = 0.052. Final scale reliability was *α* = 0.89, *ω* = 0.90. This pattern suggests that the pooled misfit likely reflects slight differences in item covariances between groups that are averaged out in the combined analysis, rather than true item‐level misspecification. Given the good fit in both subsamples and the evidence for configural and metric invariance below, this was not considered problematic.

Multi‐group CFA supported configural invariance (CFI = 0.998, RMSEA = 0.030) and metric invariance (ΔCFI = 0.001, ΔRMSEA = −0.012). Full scalar invariance was not supported (ΔCFI = −0.023, ΔRMSEA = 0.046), but partial scalar invariance was achieved by freeing the intercepts of CRS2 and CRS7 (ΔCFI = 0.001, ΔRMSEA = −0.009). Using males as the reference group, the latent mean difference for females was small and non‐significant (estimate = 0.101, SE = 0.116, *p* = 0.382), with identical magnitude but opposite sign when the reference group was reversed.

#### 2.1.2 CSJAS‐R

All 11 candidate items (CSJASR1‐11) together had *α* = 0.92 and *ω* = 0.92. The items had KMO = 0.95 and Bartlett's sphericity Χ^2^ (55) = 3536.5, *p* < 0.001, indicating an excellent start for the EFAs. The first EFA was run with all 11 candidate items and produced a unifactorial solution explaining 52.3% of variance with loadings between 0.46–0.82. Two items with loadings below 0.50 were discarded form analyzes (CSJASR10‐11). Residual correlations were then inspected in R. The initial CFA for all 9 items had CFI = 0.96, TLI = 0.95, SRMR = 0.03, Χ^2^ (27, 624) = 167.9 (*p* < 0.001), and RMSEA = 0.09 indicating modest fit. Two items (CSJASR8‐9) were then removed due to having several absolute values of residual correlations above 2.0 per item. CSJASR2 and CSJASR7 were conceptually similar and had similar residual correlations, with those of CSJASR2 slightly higher. Retaining CSJASR7 would have resulted in slightly worse residuals for the remaining model. Its inclusion would also have compromised model interpretability, as it would have led to a trivially perfect global model (CFI = 1.000, RMSEA = 0.000), which can occur in small models with low degrees of freedom. CSJASR2 was thus retained instead, which resulted in a six‐item (CSJASR1‐6) scale with excellent fit: CFI = 0.999, TLI = 0.999, SRMR = 0.012, Χ^2^ (9, 624) = 10.236 (*p* = 0.332), RMSEA = 0.015 (one residual's absolute value was 2.008, which is at the threshold). Final scale reliability was *α* = 0.88 and *ω* = 0.89.

Multi‐group CFA for the CSJAS‐R supported configural invariance (CFI = 0.999, RMSEA = 0.018) but not full metric invariance (ΔCFI = −0.010, ΔRMSEA = 0.031) or full scalar invariance (ΔCFI = −0.009, ΔRMSEA = 0.010). Partial scalar invariance was achieved by freeing the intercept of CSJAS‐R2 (ΔCFI = −0.002, ΔRMSEA = 0.000). Using males as the reference group, females had significantly higher latent means on the CSJAS‐R factor (estimate = 0.434, SE = 0.093, *p* < 0.001), with identical magnitude but opposite sign when the reference group was reversed.

### 2.2 Correlations and Descriptive Statistics

The CRS was strongly correlated with being on the right (*r* = 0.55; Table 6) on the left–right axis and conservative on the liberal‐conservative axis (*r* = 0.70). The CRS was not correlated with happiness (*r* = −0.03). The scale was weakly correlated with personal experiences of oppression (*r* = 0.20) and not correlated with finding political violence justified (*r* = −0.06). The CSJAS‐R was strongly correlated with being on the left (*r* = −0.77) on the left–right axis and liberal (*r* = −0.62) on the liberal‐conservative axis. The CSJAS‐R was very weakly negatively correlated with happiness (*r* = −0.09), but not with personal experiences of oppression (*r* = −0.01). The CSJAS‐R had a small‐to‐moderate correlation with finding political violence justified (*r* = 0.28). CRS was moderately negatively correlated with having an external locus of control (*r* = −0.39) whereas CSJAS‐R was strongly correlated with having an external locus of control (*r* = 0.60). CRS and CSJAS‐R were strongly negatively correlated (*r* = −0.62), indicating divergent validity for both scales. CSJAS‐R was nearly perfectly correlated with the original CSJAS (*r* = 0.94).

Descriptive statistics (means and standard deviations) are displayed in Table 1 for all respondents, men, women, those who responded “other” for gender, students, faculty, and people with different party affiliations. There was no meaningful difference in the CRS scores for men (1.23) and women (1.25). Percentage support for each scale item by gender are shown in Table 4. Overall, as seen in Table 5, men in the sample rejected every CRS item as did women. Men also rejected every CSJAS‐R item whereas women were in‐between about CSJASR4 and CSJASR5. Finns Party voters on average supported one CRS item, were in‐between about three of the items, and rejected one item (CRS5). CD voters were in‐between about every CRS item. Left Alliance voters supported every CSJAS‐R item. Whereas participants with a vocational degree scored on average 1.84 on the CRS, respondents with a master's degree and a doctoral degree scored only 0.86 and 0.77, respectively. This was almost reversed in that vocational degree holders scored on average only 0.93 on the CSJAS‐R but master's degree holders had 1.56 and 1.50, respectively.

**TABLE 1:** Descriptive statistics for select populations: Main variables.

	CRS (0–4)	CSJ‐R (0–4)	HA (0–10)	OP (0–4)	VIO (0–4)
All	1.22	1.29	7.83	1.11	0.51
Men	1.23	1.05	7.84	0.94	0.53
Women	1.25	1.39	7.97	1.16	0.41
Other	0.38	**3.10**	6.63	**2.38**	**1.57**
Non‐acad	1.37	1.16	7.84	1.15	0.48
Student	0.92	1.53	7.73	1.03	0.54
Faculty	0.64	1.83	8.02	1.14	0.52
Left Alliance	0.33	2.81	7.22	1.07	1.22
Greens	0.32	2.29	8.01	0.78	0.56
SDP	0.56	1.91	7.74	1.59	0.50
SPP	(0.71)	(1.15)	(7.67)	(1.13)	(0.63)
Center P	0.98	1.69	7.58	1.33	0.67
Nat Coalition	1.14	0.89	**8.25**	0.86	0.30
Christian D	1.93	0.89	8.18	1.28	0.23
Finns P	**2.26**	0.30	7.75	1.29	0.35

*Note:* Highest score **bolded**. Abbreviations: CRS, critical right scale; CSJ‐*R*, critical social justice attitude revised scale; Faculty, university faculty; HA, happiness; OP, oppression experience; Student, university student; VIO, political violence justification.

**TABLE 2:** Candidate items and factor loadings for CRS (reverse items marked ‘*r*’; altered wordings with superscript numbers).

	Item	EFA loadings	
		18‐item	7
**CRS1**	**My country's population is being replaced as part of a plan**.	**0.82**	**0.84**
**CRS2**	**Society discriminates against white people**.	**0.80**	**0.78**
**CRS3**	**A good ruler breaks rules to protect national interests**.	**0.63**	**0.64**
**CRS4**	**Conservative values should determine which expressions are allowed and which are forbidden**.	**0.62**	**0.59**
**CRS5**	**Regular people know what's good for the country better than educated experts**.	**0.68**	**0.68**
CRS6	*Each country should pursue its own interests regardless of international agreements*.	0.79	—
CRS7	*The media and education should prioritize conservative narratives*.	0.80	—
CRS8	*Globalism does not present a threat to our country. (r)*	0.61	—
CRS9	*Immigration must be significantly curtailed to preserve national unity*.	0.83	—
CRS10	*White people don't need a political movement that specifically advances the interests of white people*.	0.61	—
CRS11	*The influence Christianity has in our country should be reduced. (r)*	0.69	—
CRS12	*Liberalism has eroded the moral foundation of our society*.	0.79	—
CRS13	*Government workers who don't share the ruler's views should be removed from government*.	0.51	—
CRS14	*Taxpayer money should not be used for other countries' conflicts*.	0.70	—
CRS15	*The outcome of World War II made Germany appear worse than it was*.	0.55	—
CRS16	*Freedom of speech can't be limitless if it threatens national unity*.	−0.03	—
CRS17	*Peace should be prioritized even when allies need military support*.	0.13	—
CRS18	*A strong country is better than a weak democracy*.	0.44	—
**CSJASR1**	**If white people have on average a higher level of income than black people, it is mostly because of racism**.	**0.82**	**0.84**
**CSJASR2**	Trans* women should be allowed to compete with women in sports. (*trans woman = a person who was assigned the male sex at birth but identifies as a woman)	**0.82**	**0.80**
**CSJASR3**	**University reading lists should include fewer white or European authors**.	**0.77**	**0.77**
**CSJASR4**	***We should have more safe spaces in society (= a space from which prejudice, conflict, criticism, or potentially offensive actions, ideas, or conversations have been removed with agreed upon rules)***	**0.78**	**0.76**
**CSJASR5**	**Racism exists more in structures than in the actions of individuals**.	**0.67**	**0.67**
**CSJASR6**	***In general, we don't need to talk more about the color of people's skin. (r)***	**0.64**	**0.64**
CSJASR7	Trans* women are women. (*trans woman = a person who was assigned the male sex at birth but identifies as a woman)	0.82	—
CSJASR8	*There are two biological sexes in the human species. (r)*	0.80	—
CSJASR9	Microaggressions* should be challenged often and actively. (*microaggression = a verbal communication or behavior that can be interpreted as negative toward a minority group regardless of original intent)	0.76	—
CSJASR10	*A member of a privileged group can adopt features or cultural products of a less privileged group. (r)*	0.49	—
CSJASR11	*In general, a white person cannot understand how a black person feels equally well as another black person*.	0.46	—

*Note:* Factors forced to load on to a single factor for CRS and CSJAS‐R and a single factor for CSJAS‐R. Final scale items in bold.

**TABLE 3:** Candidate items for CRS and CSJAS‐R in original Finnish (reverse items marked ‘*r*’).

	Item
CRS1	*Kotimaani väestöä vaihdetaan suunnitellusti*.
CRS2	*Yhteiskunta syrjii valkoihoisia ihmisiä*.
CRS3	*Hyvä hallitsija rikkoo sääntöjä kansallisten etujen turvaamiseksi*.
CRS4	*Konservatiivisten arvojen tulisi määrätä mitkä ilmaisut ovat sallittuja ja mitkä kiellettyjä*.
CRS5	*Tavalliset ihmiset tietävät paremmin, mikä on maalle hyväksi, kuin koulutetut asiantuntijat*.
CRS6	*Jokaisen maan tulisi ajaa omaa etuaan riippumatta kansainvälisistä sopimuksista*.
CRS7	*Median ja koulutuksen tulisi edistää konservatiivisia narratiiveja*.
CRS8	*Globalismi ei ole uhka maallemme*.
CRS9	*Maahanmuuttoa tulee rajoittaa merkittävästi kansallisen yhtenäisyyden vuoksi*.
CRS10	*Valkoihoiset eivät tarvitse erikseen heidän etujaan ajavaa poliittista liikettä*.
CRS11	*Kristinuskon vaikutusvaltaa tulisi vähentää maassamme*.
CRS12	*Liberalismi on rapauttanut yhteiskunnan moraalisen perustan*.
CRS13	*Hallinnosta tulisi poistaa virkamiehet, joilla on eri mielipiteet kuin hallitsijalla*.
CRS14	*Veronmaksajien rahoja ei tulisi käyttää muiden maiden konflikteihin*
CRS15	*Toisen maailmansodan lopputulos sai Saksan näyttämään huonommalta kuin se oli*.
CRS16	*Sanavapaus ei voi olla rajoittamatonta, jos se uhkaa kansallista yhtenäisyyttä*.
CRS17	*Rauha tulisi asettaa etusijalle myös silloin, kun liittolaiset vaativat sotilaallista tukea*
CRS18	*Vahva valtio on parempi kuin heikko demokratia*.
CSJASR1	*Tuloerot valkoihoisten ja tummaihoisten ihmisten välillä selittyvät enimmäkseen rasismilla*.
CSJASR2	*Transnaisten tulisi saada osallistua urheilukilpailuissa naisten sarjaan. (transnainen = syntymässä pojaksi määritelty henkilö, joka identifioituu naiseksi)*
CSJASR3	*Korkeakoulujen kurssikirjallisuuden tulisi sisältää vähemmän valkoihoisia tai eurooppalaisia kirjailijoita*.
CSJASR4	*Yhteiskunnassa tulisi olla enemmän turvallisempia tiloja. (= tila, josta on sovituin säännöin pyritty poistamaan ennakkoluulot, konflikti, kritiikki tai potentiaalisesti loukkaavat teot, ideat tai keskustelut)*.
CSJASR5	*Rasismi on enemmän rakenteellinen kuin yksilöiden toiminnassa näkyvä ilmiö*.
CSJASR6	*Ihonväriin keskittyminen ei yleisesti ottaen ole tarpeellista ihmisoikeuksien edistämiseksi*.
CSJASR7	*Transnaiset ovat naisia. (transnainen = syntymässä pojaksi määritelty henkilö, joka identifioituu naiseksi)*
CSJASR8	*Ihmislajilla on kaksi biologista sukupuolta*.
CSJASR9	*Mikroaggressioihin tulee puuttua usein ja aktiivisesti. (mikroaggressio = sanallinen viesti tai käytös, jonka voi tulkita viestivän kielteisiä asenteita vähemmistöryhmää kohtaan, riippumatta alkuperäisestä tarkoituksesta)*
CSJASR10	*Etuoikeutetun ryhmän jäsen saa ottaa käyttöönsä vähemmän etuoikeutetun ryhmän piirteitä tai kulttuurituotteita*.
CSJASR11	*Valkoihoinen ihminen ei yleisesti ottaen voi ymmärtää tummaihoista ihmistä yhtä hyvin kuin toinen tummaihoinen ihminen*.

**TABLE 4:** CRS and CSJAS‐R item answer distributions in percentages (all items unreversed; reverse items marked ‘*r*’).

	Item	Men (%)					Women (%)				
		1	2	3	4	5	1	2	3	4	5
**CRS1**	**My country's population is being replaced as part of a plan**.	**51.8**	**13.2**	**6.8**	**20.3**	**8.0**	**49.0**	**11.5**	**13.4**	**15.0**	**11.1**
**CRS2**	**Society discriminates against white people**.	**40.6**	**17.3**	**12.8**	**22.4**	**7.0**	**49.6**	**18.9**	**9.8**	**16.7**	**4.9**
**CRS3**	**A good ruler breaks rules to protect national interests**.	**30.5**	**31.5**	**9.4**	**21.8**	**6.8**	**29.2**	**31.1**	**12.5**	**21.4**	**5.8**
**CRS4**	***Conservative values should determine which expressions are …***	**58.8**	**21.2**	**8.2**	**8.5**	**3.3**	**52.3**	**22.7**	**12.7**	**10.4**	**1.9**
**CRS5**	***Regular people know what's good for the country better than …***	**27.1**	**38.4**	**14.8**	**16.1**	**3.5**	**20.6**	**36.7**	**21.3**	**16.9**	**4.5**
CRS6	*Each country should pursue its own interests regardless of …*	26.0	25.3	8.0	29.5	11.2	26.9	27.2	5.6	25.4	14.9
CRS7	*The media and education should prioritize conservative narratives*.	36.7	18.5	20.8	17.2	6.8	35.9	14.5	19.1	23.3	7.3
CRS8	*Globalism does not present a threat to our country. (r)*	15.0	28.7	10.4	29.0	16.9	20.5	27.7	14.8	25.8	11.4
CRS9	*Immigration must be significantly curtailed to preserve national unity*.	22.2	18.7	6.7	21.9	30.5	22.7	17.8	8.2	24.9	26.4
CRS10	*White people don't need a political movement that specifically … (r)*	15.7	17.6	13.4	20.3	33.0	11.2	13.5	21.2	20.0	34.2
CRS11	*The influence Christianity has in our country should be reduced. (r)*	32.5	19.1	17.5	13.1	17.8	42.2	18.5	11.9	12.6	14.8
CRS12	*Liberalism has eroded the moral foundation of our society*.	32.2	20.6	7.1	25.4	14.8	35.0	13.1	7.7	24.6	19.6
CRS13	*Government workers who don't share the ruler's views should be …*	63.0	24.4	4.9	5.2	2.6	73.0	18.7	5.6	2.2	0.4
CRS14	*Taxpayer money should not be used for other countries' conflicts*.	23.8	33.0	12.4	19.5	11.3	14.6	38.3	15.0	18.8	13.3
CRS15	*The outcome of World War II made Germany appear worse than it was*.	37.4	24.5	12.6	17.3	8.2	45.5	21.7	18.7	11.9	2.1
CRS16	*Freedom of speech can't be limitless if it threatens national unity*.	32.7	36.6	9.2	15.2	6.3	26.5	39.6	14.3	16.3	3.3
CRS17	*Peace should be prioritized even when allies need military support*.	11.8	31.6	19.4	26.2	11.0	3.4	16.3	22.1	33.7	24.5
CRS18	*A strong country is better than a weak democracy*.	25.9	35.0	13.9	17.0	8.2	21.7	35.7	20.0	16.5	6.1
**CSJASR1**	***If white people have on average a higher level of income than black …***	**49.5**	**28.2**	**4.6**	**10.8**	**6.9**	**29.9**	**32.6**	**9.6**	**23.4**	**4.6**
**CSJASR2**	Trans* women should be allowed to compete with women in sports. …	**70.6**	**11.9**	**5.3**	**5.0**	**7.3**	**62.9**	**13.1**	**10.0**	**8.4**	**5.6**
**CSJASR3**	***University reading lists should include fewer white or European …***	**53.5**	**21.0**	**17.7**	**5.2**	**2.6**	**36.4**	**18.2**	**26.0**	**14.3**	**5.0**
**CSJASR4**	***We should have more safe spaces in society (= a space where …)***	**41.2**	**21.5**	**8.0**	**19.0**	**10.3**	**30.5**	**18.0**	**16.5**	**19.2**	**15.8**
**CSJASR5**	**Racism exists more in structures than in the actions of individuals**.	**28.6**	**33.9**	**15.3**	**17.9**	**4.3**	**17.9**	**33.6**	**16.4**	**28.2**	**3.8**
**CSJASR6**	***In general, we don't need to talk more about the color of people's … (r)***	**5.5**	**14.8**	**4.2**	**25.8**	**49.7**	**7.6**	**17.9**	**8.0**	**27.8**	**38.8**
CSJASR7	Trans* women are women. (* = born male, identify as female)	46.3	16.9	8.1	12.1	16.6	43.0	12.1	7.4	14.1	23.4
CSJASR8	*There are two biological sexes in the human species. (r)*	8.4	10.9	3.2	10.9	66.6	11.6	14.2	2.2	14.2	57.7
CSJASR9	Microaggressions* *should be challenged often and actively. …*	39.6	21.7	8.6	21.1	8.9	21.6	19.3	11.7	28.4	18.9
CSJASR10	*A member of a privileged group can adopt features or cultural … (r)*	10.0	12.0	14.7	25.8	37.5	11.5	19.4	24.5	27.3	17.4
CSJASR11	*A white person cannot understand how a black person feels equally …*	19.8	30.4	9.9	32.0	7.9	12.6	28.7	13.8	34.1	10.7

*Note:* 1 = “completely disagree”, 2 = “somewhat disagree”, 3 = “not agree, not disagree”, 4 = “somewhat agree”, 5 = “completely agree”. Final scale items in bold.

**TABLE 5:** Agree/in‐between/reject CRS and CSJAS‐R items by participant group (reverse items reversed and marked ‘*r*’).

	Item	M		W		LA		G		NC		CD		F	
CRS1	*My country's population is being replaced as part of a plan*.	−		−		−		−		−		+/−		+	
CRS2	*Society discriminates against white people*.	−		−		−		−		−		+/−		+/−	
CRS3	*A good ruler breaks rules to protect national interests*.	−		−		−		−		−		+/−		+/−	
CRS4	*Conservative values should determine which expressions are …*	−		−		−		−		−		+/−		−	
CRS5	*Regular people know what's good for the country better than …*	−		−		−		−		−		+/−		+/−	
CSJAS‐R1	*If white people have on average a higher level of income than …*		−		−		+		+/−		−		−		−
CSJAS‐R2	Trans* women should be allowed to compete with women in …		−		−		+		+/−		−		−		−
CSJAS‐R3	*University reading lists should include fewer white or …*		−		−		+		+/−		−		−		−
CSJAS‐R4	*We should have more safe spaces in society (= a space where …)*		−		+/−		+		+		−		−		−
CSJAS‐R5	*Racism exists more in structures than in the actions of individuals*.		−		+/−		+		+/−		−		−		−
CSJAS‐R6	*In general, we don't need to talk more about the color of … (r)*		−		−		+		+/−		−		−		−
	Agreeing with items, total	0	0	0	0	0	6	0	1	0	0	0	0	1	0
	In‐between, total	1	0	1	2	0	0	0	5	1	0	5	0	3	0
	Rejection of items, total	4	6	4	4	5	0	5	0	4	6	0	6	1	6

*Note:* Agreeing: on a range 0–4, subsample average ≥ 2.50. In‐between subsample average ∈ [1.50, 2.49]. Rejection: subsample average ≤ 2.50. Abbreviations: CD, Christian democrats; F, Finns party; G, greens; LA, left alliance; M, men; NC, National coalition; W, women.

**TABLE 6:** Interitem CRS correlations and correlations with other study variables.

	C1	C2	C3	C4	C5	CRS	CSJ	LR	LC	HA	OP	VIO	GS	AGE	EL
C1		0.69	0.54	0.47	0.56	0.86	−0.56	0.49	0.58	−0.06	0.22	−0.05	−0.48	0.14	−0.32
C2			0.50	0.44	0.50	0.82	−0.60	0.52	0.58	−0.01	0.17	−0.05	−0.50	0.14	−0.37
C3				0.39	0.42	0.74	−0.41	0.38	0.44	−0.04	0.09	−0.02	−0.38	0.16	−0.28
C4					0.46	0.69	−0.35	0.40	0.57	0.04	0.18	−0.01	−0.34	0.20	−0.26
C5						0.75	−0.44	0.37	0.54	0.03	0.13	−0.07	−0.42	0.25	−0.27
CRS							−0.62	0.55	0.70	−0.03	0.20	−0.06	−0.55	0.23	−0.39
CSJ								−0.77	−0.66	−0.09	−0.01	0.28	0.76	−0.15	0.60
LR									0.62	0.16	−0.03	−0.28	−0.68	0.10	−0.58
LC										0.07	0.15	−0.15	−0.62	0.30	−0.43
HA											−0.32	−0.13	−0.08	0.09	−0.23
OP												0.05	−0.05	0.06	0.12
VIO													0.19	−0.09	0.26
GS														−0.12	0.50
AGE															−0.08
EL															

Abbreviations: AGE, age; C1, CRS item number 1 etc.; CSJ, CSJAS‐R; EL, external locus of control; GS, global CSJA; HA, happiness; LC, liberal‐conservative; LR, left–right; OP, oppression experience; VIO, violence justification.

**TABLE 7:** Descriptive statistics for select subpopulations: Select secondary variables.

	CRS %	CSJ‐R %	GS (0–4)	EL (0–4)	EU (0–4)	NATO (0–4)	TRU (0–4)	STU (0–4)	WOM	VIO	SEX	UKR NATO	SOL	GEN
All	28%	28%	1.46	0.97	2.31	2.71	0.95	2.47	4.2%	11%	73%	64%	2S 57%	46%
Men	29%	21%	1.29	0.84	2.35	2.96	1.01	2.62	5.2%	11%	78%	64%	2S 60%	45%
Men < 40	23%	27%	1.30	0.91	2.54	3.06	0.95	2.67	7.9%	15%	75%	68%	2S 73%	53%
Men > 40	33%	18%	1.27	0.79	2.23	2.90	1.06	2.57	3.5%	9%	79%	62%	2S 52%	39%
Wom	28%	30%	1.51	0.94	2.23	2.50	0.95	2.45	2.4%	8%	72%	63%	2S 55%	44%
Wom < 40	11%	44%	2.06	1.18	2.65	2.63	0.51	2.54	1.4%	8%	63%	68%	2S 84%	58%
Wom > 40	34%	25%	1.31	0.85	2.10	2.47	1.10	2.44	2.3%	7%	75%	62%	2S 44%	39%
Oth gender	6%	**94%**	3.38	**2.33**	2.88	1.57	0.00	1.25	0%	**43%**	25%	69%	2S 53%	94%
Left All.	0%	84%	3.31	2.20	2.90	1.75	0.05	1.54	1.3%	31%	26%	81%	2S 77%	95%
Left All. M	0%	72%	2.97	1.97	2.95	1.85	0.03	1.69	2.6%	40%	33%	77%	2S 79%	95%
Left All. W	0%	92%	**3.70**	2.08	2.92	2.04	0.12	1.88	0%	12%	26%	**95%**	2S 95%	92%
Greens	2%	77%	2.95	1.45	3.32	2.64	0.07	2.48	1.6%	9%	33%	77%	2S 92%	91%
Greens M	0%	69%	2.96	1.48	**3.52**	2.89	0.07	2.62	0%	7%	31%	79%	2S 89%	90%
Greens W	0%	83%	3.06	1.31	3.23	2.38	0.03	2.38	0%	9%	32%	76%	**2S100%**	**97%**
SDP	9%	53%	2.45	1.76	2.97	2.53	0.29	2.24	0%	9%	46%	81%	2S 94%	81%
Nat. Coal.	20%	8%	0.91	0.45	2.82	3.48	0.86	3.36	5.8%	5%	88%	70%	2S 65%	34%
Nat C. M	22%	6%	0.80	0.36	2.78	**3.58**	1.01	**3.39**	7.3%	6%	92%	69%	2S 66%	34%
Nat. C. W	15%	12%	1.04	0.63	2.90	3.32	0.61	3.34	3.5%	4%	79%	71%	2S 64%	32%
ChristD	55%	4%	0.41	0.56	1.33	2.13	1.84	2.33	1.9%	4%	**100%**	49%	I 59%	4%
Finns P	66%	0%	0.35	0.38	1.06	2.76	**2.16**	2.06	7.0%	8%	99%	45%	I 59%	6%
Finns P M	**67%**	0%	0.35	0.42	1.09	2.92	2.13	2.17	**8.9%**	9%	**100%**	50%	I 59%	6%
Finns P W	63%	0%	0.35	0.31	1.00	2.47	2.23	1.86	3.3%	6%	97%	33%	I 59%	6%

*Note:* Table 6 abbreviations & CRS and CSJ‐R %: % on average supporting, TRU = Trump, STU=Stubb, WOM=“Women can deserve violence … because … dress, behavior, or appearance.” SEX = % “there are two biol sexes”, VIO = % not opposed to political violence, UKR NATO = % support Ukraine Nato membership, SOL = preferred solution to Israel‐Palestine conflict (2 = two state solution; I = single state, Israel; *P* = single state, Palestine), GEN = % “Israel has committed genocide in Gaza”. () = *n* < 10. Largest in **bold**.

A binary variable for CRS scores below (*n* = 421) and above (*n* = 199) the scale midpoint (2.0) was constructed for classifying the sample into those in opposition to and those in support of the average scale item. As seen in Table 7, 28% of the study sample were more in support of the average CRS item than not, 29% for men, 28% for women. For CSJAS‐R the corresponding number was 28% for the sample, 21% for men and 30% for women. The highest support for CRS was in the small group (*n* = 13) of people who don't vote with 77%, Finns Party voters with 66% and CD voters with 55% of people supporting the average CRS item. Lowest support was seen in Left Alliance and Greens voters with 1% and 2%, respectively. For CSJAS‐R the highest support was in Left Alliance voters and Greens voters with 84% and 77% support. The lowest was in Finns Party and CD voters with 0% and 4%.

### 2.3 Secondary Variables

Numerous secondary variables were used to evaluate participant approval and disapproval of political ideologies, famous people, and current events (see Table 7 for more details). Study participant groups were nearly universally strongly in support of democracy (ranging from 3.40–3.88 on a scale of 0–4). Participant groups also expressed strong disapproval for Vladimir Putin (0.00–0.29, range: 0–4) and Hamas (0.00–0.76, 0–4) Support for Israel and its actions in the war following 7th of October, 2023 varied strongly between participant groups. Most Christian Democrats (CD) and Finns Party voters indicated they would prefer a one state solution with just Israel. Only 6% of CD voters wanted a two‐state solution. Taken together, approximately 5% of Finns Party and CD supporters considered Israel's actions in the war to amount to genocide. More than 90% of Greens supporters considered Israel's actions a genocide and preferred a two‐state solution. 18% of Left Alliance supporting men and 33% of people with “other” gender preferred a single state solution with just Palestine.

This study replicated a question from a report by NYTKIS (2024) on violence against women, where in their sample 25% of men under 35 years of age said violence against women was justified based on how they “dress, act, or look like”. Numbers were considerably lower in this study: 7.9% for men under 40. However, men aged 18–24 reported 10.3% approval and men aged 25–29 14.3% approval. Taken together, support for the claim in men aged 18–29 was 11.9%. Only 1% of Left Alliance voters approved of the claim. However, almost a third were not opposed to violence against “politically dangerous” people.

The percentage of people who said there are two sexes in the human species varied strongly from 26% in Left Alliance and 33% in Greens voters to 88% in National Coalition Party and 100% in Finns Party and CD voters. Finns Party and CD voters were hesitant about letting Ukraine into NATO with 45% and 49% respective support whereas Left Alliance and Greens were the most welcoming with 81% and 77%. Left‐wing party voters expressed the highest external locus of control whereas numbers were low in CD, National Coalition, and Finns Party voters.

## 3 Discussion

This study aimed to design and evaluate a scale for assessing critical right attitudes. The resulting Critical Right Scale (CRS) showed strong psychometric properties, including good model fit and reliability. CRS scores were weakly positively correlated with reported experiences of oppression. The scale was negatively associated with critical social justice attitudes as well as external locus of control. In terms of party affiliation, critical right attitudes were most prevalent among voters of the Finns Party and Christian Democrats, and virtually absent among supporters of the Left Alliance and Greens. The final unidimensional scale consisted of five items reflecting the three theorized facets of the critical right: identitarian grievance (CRS1, CRS2), postmodern conservatism (CRS4, CRS5), and authoritarian nationalism (CRS3 and CRS4, the latter spanning both of these latter facets). They also align with two broader components: identity hierarchies (CRS1–CRS3, CRS5) and speech regulation (CRS4). In the present data, identitarian grievance items were endorsed mainly by Finns Party and Christian Democrat voters, suggesting concentrated, not broadly distributed, grievance on the right. Postmodern conservatism and authoritarian nationalism items indicated that speech regulation on the right is tied to national identity and authority, in contrast to harm‐prevention norms often invoked on the left. The absence of a CRS–violence link, versus a small‐to‐moderate positive association for CSJAS‐R, suggests distinct behavioral correlates despite shared structural features.

A second aim of the study was to replicate and improve the Critical Social Justice Attitudes Scale. Factor analyzes and residual comparisons supported a revised six‐item version (CSJAS‐R), which replaced two original items (standpoint theory, cultural appropriation) with a new item on structural racism. The revised scale showed excellent psychometric performance and was highly correlated with participants' self‐assessed identification with “woke” attitudes. It was also strongly associated with external locus of control and modestly with justification of political violence. Politically, elevated CSJAS‐R scores were found among supporters of the Greens and Left Alliance.

While both scales showed excellent fit in their respective subsamples, the CRS pooled model retained a small number of residual correlations above the |2.0| threshold. Given the good fit in men and women separately and evidence for configural and metric invariance for the CRS, this misfit likely reflects minor group‐level covariance differences rather than substantive misspecification. Invariance testing supported only partial scalar invariance for both CRS and CSJAS‐R, which is generally sufficient for comparing latent means but should be noted in interpreting group differences (Chen 2007). Specifically, regarding the CSJAS‐R, while the change in fit indices marginally failed the strict metric invariance criterion, the resulting model provided excellent fit and the achievement of partial scalar invariance supported the validity of latent mean comparisons.

The results also underscore the extent of ideological polarization. Support for the average CRS item was concentrated among Finns Party and Christian Democrat voters, and almost entirely absent among Left Alliance and Green voters. Conversely, CSJAS‐R support was highest among Left Alliance and Greens, with negligible endorsement among Finns Party and Christian Democrats. These patterns indicate that the “critical” orientations measured here are closely tied to party alignment, with limited overlap between constituencies. Despite their common emphasis on identity‐based grievance and speech regulation, the two scales differ in correlates such as justification of political violence, highlighting important ideological divergences.

Public commentary suggests that both ideological styles are likely to persist, although their specific manifestations may evolve (McWhorter 2025a). Critical right politics in particular remain in an emergent phase, and the CRS represents an initial attempt to operationalize this construct. Some theorists have proposed that the far left and far right are best understood through a “horseshoe” model, in which the two extremes closely resemble each other despite opposite starting points (Taylor 2006). The present findings lend partial support to this idea. One promising future direction might be to develop a unified “critical politics” scale encompassing both left‐wing and right‐wing variants.

Taken together, the inclusion of both scales in a single study provides a rare opportunity for direct empirical comparison of left‐ and right‐oriented “critical” politics. Their strong psychometric performance suggests utility for cross‐national and longitudinal research on ideological polarization. The differing associations with political violence also point to a potentially important domain for future investigation.

### 3.1 Limitations

This study has several limitations. First, the sample size, while adequate, could have been larger and more representative of the Finnish population. Notably, supporters of the Social Democratic Party and Center Party were underrepresented. Recruitment relied on university and personal networks as well as social media accounts of politicians and party activists, potentially introducing bias toward politically engaged or digitally active participants. Also, as the sample came from politically distinct digital “pockets,” study subgroups (like women over 40 years old) can be overly biased toward certain ideologies. Given the self‐selection bias and deliberate oversampling of right‐wing respondents, the sample is non‐random and cannot be considered representative; therefore, claims about the Finnish population at large should not be made from these findings.

Second, the concept of the critical right is novel and lacks prior validated instruments. The term “woke right” only entered mainstream discourse in late 2024 and early 2025 (Chatterton‐Williams 2025; Kelly 2025; McWhorter 2025a; The Economist 2025), requiring the study to adopt an exploratory framework. No established convergent validity criterion was available, unlike the CSJAS‐R, which used self‐reported identification with “wokeness” as a benchmark.

Third, as most discourse around critical right politics may originate in anglophone countries (particularly the United States) a Finnish sample may be suboptimal for capturing the construct in its cultural context. However, prior reporting and research suggest that anglophone identity politics have become widespread across Northern Europe, including Finland (Boztas 2022; Lahtinen (2024); Koskela 2021; Sande 2023). Fourth, both ideological styles examined here are evolving. Item performance, especially for the CSJAS, has shown variation across samples. Replication and scale refinement should continue as cultural salience and discourse change.

## Author Contribution

**Oskari Lahtinen:** designed the study, collected the data, conducted the analyzes, and wrote the manuscript.

## Funding

The author has nothing to report.

## Consent

Informed consent to participate in the study was obtained from all subjects.

## Conflicts of Interest Statement

The author has been affiliated with the Greens and National Coalition Party and has participated in local politics as a Greens member.

## Data Availability

The data that support the findings of this study are openly available in OSF at https://osf.io/9ux6m/files/osfstorage.

## References

[sjop70070-bib-0001] Boztas, S. 2022. “Justice Minister Slams Wokeism, Extreme Right and Conspiracy Theorists in Democracy Speech.” *Dutch News*, September 13. Accessed 5 August 2025.

[sjop70070-bib-0002] Buchanan, P. J. 2011. Suicide of a Superpower: Will America Survive to 2025? Macmillan.

[sjop70070-bib-0003] Chatterton‐Williams, T. 2025. “How the Woke Right Replaced the Woke Left.” *The Atlantic*, February 17. https://www.theatlantic.com/ideas/archive/2025/02/woke‐right/681716/.

[sjop70070-bib-0004] Chen, F. F. 2007. “Sensitivity of Goodness of Fit Indexes to Lack of Measurement Invariance.” Structural Equation Modeling: A Multidisciplinary Journal 14, no. 3: 464–504.

[sjop70070-bib-0005] DeYoung, K. 2022. The Rise of Right‐Wing Wokeism. Gospel Coalition.

[sjop70070-bib-0025] Duckitt, J. , and C. G. Sibley . 2010. “Personality, Ideology, Prejudice, and Politics: A Dual‐Process Motivational Model.” Journal of Personality 78, no. 6: 1861–1894. 21039534 10.1111/j.1467-6494.2010.00672.x

[sjop70070-bib-0006] EVA/Elinkeinoelämän valtuuskunta . 2025. “Toinen askel oikealle” Accessed 5 August 2025. https://www.eva.fi/wp‐content/uploads/2025/07/eva‐analyysi‐no‐151.pdf.

[sjop70070-bib-0007] Graham, J. , J. Haidt , S. Koleva , et al. 2013. “Moral Foundations Theory: The Pragmatic Validity of Moral Pluralism.” In Advances in Experimental Social Psychology, vol. 47, 55–130. Academic Press.

[sjop70070-bib-0008] Helliwell, J. F. , R. Layard , J. Sachs , and J. E. De Neve . 2020. “World Happiness Report 2020.”

[sjop70070-bib-0009] Kelly, J. 2025. “The Scourge of the ‘Woke Right’.” *The Financial Times*, May 17. Accessed 5 August 2025. https://www.ft.com/content/546040d5‐91b6‐46e9‐9479‐2ce463d76cb8.

[sjop70070-bib-0010] Koskela, M. 2021. “Ylen Marja Sannikka ‐ohjelma herätti keskustelun cancel‐kulttuurista, wokesta ja callouttaamisesta – lue tästä, mistä termeissä on kyse.” *Yle*, November 23. Accessed 5 August 2025. https://yle.fi/a/3‐12199882.

[sjop70070-bib-0024] Lahtinen, O. 2024. “Construction and Validation of a Scale for Assessing Critical Social Justice Attitudes.” Scandinavian Journal of Psychology 65, no. 4: 693–705. 38482728 10.1111/sjop.13018

[sjop70070-bib-0011] Lawler, P. A. 2002. “Conservative Postmodernism, Postmodern Conservatism.” Intercollegiate Review 38, no. 1: 16.

[sjop70070-bib-0012] McManus, M. 2019. The Rise of Post‐Modern Conservatism: Neoliberalism, Post‐Modern Culture, and Reactionary Politics. Springer Nature.

[sjop70070-bib-0013] McWhorter, J. 2025a. “How ‘Woke’ Became the ‘Woke Right’ (And Why It Shouldn't Surprise Anyone).” *The New York Times*, February 20. Accessed 5 August 2025.

[sjop70070-bib-0014] McWhorter, J. 2025b. “Wokeness Will Always Be With Us.” *The New York Times*, July 3. Accessed 5 August 2025. https://www.nytimes.com/2025/07/03/opinion/wokeness‐race‐gaza.html.

[sjop70070-bib-0015] Mounk, Y. 2023. The Identity Trap: A Story of Ideas and Power in Our Time. Penguin.

[sjop70070-bib-0016] Mutz, D. C. 2018. “Status Threat, Not Economic Hardship, Explains the 2016 Presidential Vote.” Proceedings of the National Academy of Sciences 115, no. 19: E4330–E4339. 10.1073/pnas.1718155115 PMC5948965 29686081

[sjop70070-bib-0017] NYTKIS ry/Naisjärjestöt yhteistyössä . 2024. Ei kaikki miehet – mutta liian moni Accessed 5 August 2025. https://nytkis.org/vakivaltatutkimus‐artikkeli‐2024/.

[sjop70070-bib-0018] Pelkonen, L. 2025. “Ylen kannatusmittaus: Perussuomalaiset menettää äänestäjiä joka suuntaan – ‘Romahduksesta voidaan jo puhua’.” *YLE*, May 8. Accessed 5 August 2025. https://yle.fi/a/74‐20160212.

[sjop70070-bib-0019] Sande, B. 2023. “Därför är Stockholmare mer woke än lantisar.” *Fokus* .

[sjop70070-bib-0020] Schmitt, C. 1932. The Concept of the Political. University of Chicago Press.

[sjop70070-bib-0021] Sensoy, O. , and R. DiAngelo . 2017. Is Everyone Really Equal?: An Introduction to Key Concepts in Social Justice Education. Teachers College Press.

[sjop70070-bib-0022] Taylor, J. 2006. Where Did the Party Go?: William Jennings Bryan, Hubert Humphrey, and the Jeffersonian Legacy. University of Missouri Press.

[sjop70070-bib-0023] The Economist . 2025. “Does America Now Have a Woke Right?” *The Economist*, June 10 Accessed 5 August 2025. https://www.economist.com/united‐states/2025/06/10/does‐america‐now‐have‐a‐woke‐right.

